# Prenatal Diagnosis and Genetic Analysis of 21q21.1–q21.2 Aberrations in Seven Chinese Pedigrees

**DOI:** 10.3389/fgene.2021.731815

**Published:** 2021-12-21

**Authors:** Huamei Hu, Rong Zhang, Yongyi Ma, Yanmei Luo, Yan Pan, Juchun Xu, Lupin Jiang, Dan Wang

**Affiliations:** Department of Gynecology and Obstetrics, Southwest Hospital, Third Military Medical University (Army Medical University), Chongqing, China

**Keywords:** 21q21.1–21.2 duplication, 21q21.1–21.2 deletion, SNP array, NCAM2, prenatal diagnosis

## Abstract

**Background:** Chromosomal aberrations contribute to human phenotypic diversity and disease susceptibility, but it is difficult to assess their pathogenic effects in the clinic. Therefore, it is of great value to report new cases of chromosomal aberrations associated with normal phenotypes or clinical abnormalities.

**Methods:** This was a retrospective analysis of seven pedigrees that carried 21q21.1–q21.2 aberrations. G-banding and single-nucleotide polymorphism array techniques were used to analyze chromosomal karyotypes and copy number variations in the fetuses and their family members.

**Results:** All fetuses and their family members showed normal karyotypes in seven pedigrees. Here, it was revealed that six fetuses carried maternally inherited 21q21.1–q21.2 duplications, ranging from 1 to 2.7 Mb, but none of the mothers had an abnormal phenotype. In one fetus, an 8.7 Mb deletion of 21q21.1–q21.2 was found. An analysis of the pedigree showed that the deletion was also observed in the mother, brother, and maternal grandmother, but no abnormal phenotypes were found.

**Conclusion:** This study identified 21q21.1–q21.2 aberrations in Chinese pedigrees. The carriers of 21q21.1–q21.2 duplications had no clinical consequences based on their phenotypes, and the 21q21.1–q21.2 deletion was transmitted through three generations of normal individuals. This provides benign clinical evidence for pathogenic assessment of 21q21.1–q21.2 duplication and deletion, which was considered a variant of uncertain significance and a likely pathogenic variant in previous reports.

## Introduction

To date, the 21q21 duplication and deletion have not been included in the known pathogenicity syndromes. However, early in the 1980s, Park et al. reported that partial trisomy of chromosome 21, comprising the *NCAM2* gene, results in intellectual disability but does not cause other phenotypes of Down syndrome (DS) (Park et al., 1987). Haldeman-Englert et al. revealed that a boy who was evaluated for autistic features, significant speech delay, and poor social interactions carried a *de novo* 8.8 Mb 21q21.1–q21.3 deletion involving the *NCAM2* gene (Haldeman-Englert et al., 2009). In addition, three cases of neurodevelopmental disorders were reported, with clinical phenotypic abnormalities including global developmental delay, behavioral disorders, and impaired social interactions. All of them carried 21q21.1–21.2 deletions involving *NCAM2* (Petit et al., 2015). Another case report revealed that a boy with autism spectrum disorder and macrocephaly carried a 1.6 Mb deletion of 21q21.1–21.2, containing the *NCAM2* gene, but no other functional gene (Scholz et al., 2016). Previously, *NCAM2* was proposed as a candidate gene for autism based on genome-wide association studies (Hussman et al., 2011). Duplications, deletions, and single-nucleotide polymorphisms of the *NCAM2* gene have been found in individuals with intellectual disabilities or autism, and these studies suggest that *NCAM2* might play a role in neurodevelopmental disorders.

In our study, the carriers of 21q21.1–21.2 duplications in six pedigrees (the region of one pedigree contained *NCAM2*) showed normal phenotypes. We further identified a rare 8.7 Mb deletion of 21q21.1–21.2 containing *NCAM2*, which had been transmitted through three generations of normal individuals. These findings provide benign evidence, which is important for accurate genetic counseling on 21q21.1–21.2 aberrations in prenatal diagnosis.

## Materials and Methods

### Subjects

A retrospective study was performed from January 2016 to December 2020. In total, seven cases carrying 21q21.1–21.2 deletions or duplications were selected from 11,867 pregnant women who had indications (e.g., abnormal non-invasive prenatal testing (NIPT) or fetal imaging) and underwent invasive diagnostic testing *via* amniocentesis or cordocentesis at the Prenatal Diagnosis Center of Obstetrics and Gynecology, Southwest Hospital. Informed consent for invasive prenatal diagnosis was obtained from the parents before detection. This research was approved by the Ethics Committee of Southwest Hospital, Third Military Medical University (Army Medical University). Six fetuses from six unrelated Chinese families were identified as carrying 21q21.1–q21.2 duplications, as their pedigree verification information was collected, and they were classified as pedigrees 1, 2, 3, 4, 5, and 6. In addition, a fetus carrying a 21q21.1–q21.2 deletion and its family members were labeled as pedigree 7. The pregnant women in these seven pedigrees did not have pregnancy complications and denied any related family history.

Pedigrees 1–6: The maternal age at the time of amniocentesis was between 25 and 32 years, and a gestational age ranging from 18 + 2 to 25 weeks. The pregnant woman in pedigree 3 chose amniocentesis because of pulmonary sequestration of the fetus examined by ultrasound. The others all chose amniocentesis because NIPT screening showed an abnormality on chromosome 21.

Pedigree 7: A 22-year-old woman (gravida 4, para 1) was subjected to cordocentesis at 26 + 5 gestational weeks because the bilateral ventricle of the fetus had widened, as tested by ultrasound examination (left: 14 mm, right: 14 mm).

### Chromosomal Karyotyping

Approximately 0.5 ml of each peripheral blood sample and 0.4 ml of each umbilical cord blood sample were inoculated into a T-cell culture medium (BAIDI, China) and incubated at 37°C, for 3 days. Approximately 20 ml of each amniotic fluid sample was inoculated into an amniotic fluid medium (BIO-AMF™-2, BI, China) and incubated at 37°C, with 5% CO_2_, for 7–10 days. Chromosomal karyotyping was performed according to the standard protocol using G-banding at a 400-banded (amniotic fluid samples) or 550-banded (blood samples) resolution, and karyotypes were described according to the International System for Human Cytogenetic Nomenclature 2016 (ISCN 2016) criteria (Stevens-Kroef et al., 2017).

### Single-Nucleotide Polymorphisms Array Analysis

Uncultured amniotic fluid samples (10 ml per fetus), umbilical cord blood samples (600 µL per fetus), and peripheral blood (600 µL per person) of the pedigree members were collected, and DNA was extracted using the TIANamp Genomic DNA Kit (TIANGEN, China). The Infinium Global Screening Array (Illumina, San Diego, CA, United States) contains approximately 700,000 genome-wide tag SNPs. Genomic DNA was hybridized to an Infinium Global Screening Array as reported previously (Srebniak et al., 2011). The array was scanned with the iScan array scanning system (Illumina, San Diego, CA, United States). Molecular karyotype analysis was performed using GenomeStudio V2011.1 software (Illumina, San Diego, CA, United States), which was used for annotation. Copy number variations (CNVs) that were larger than 100 kb or affected more than 50 markers were considered and were annotated based on the GRCh37 (hg19) genome. CNVs were evaluated according to the guidelines (Richards et al., 2015; Riggs et al., 2020), scientific literature, and publicly available databases as follows: DGV (http://dgv.tcag.ca/dgv/app/home), OMIM (http://www.ncbi.nlm.nih.gov/omim), gnomAD (http://gnomad-sg.org/), DECIPHER (http://decipher.sanger.ac.uk), dbVar (http://www.ncbi.nlm.nih.gov/dbvar), ClinVar(http://www.ncbi.nlm.nih.gov/clinvar), ClinGene (https://www.ncbi.nlm.nih.gov/projects/dbvar/clingen/), and Pubmed (https://www.ncbi.nlm.nih.gov/pubmed/). Benign or likely benign CNVs were not reported.

### Prenatal and Postnatal Follow-Up Assessment

Ultrasound results of the second and third trimesters of pregnancy were collected. Postnatal clinical follow-up assessments *via* telephone were performed from 6 months to 3 years after birth. After obtaining their parents’ informed consent, the child’s healthcare data were collected to assess developmental details. General child healthcare was carried out by professional doctors according to the World Health Organization’s physical and mental development table for infants aged 0–3 years. Child healthcare in tertiary hospitals was performed according to the Denver Developmental Screening Test (Wijedasa, 2012).

## Results

### Analysis of the Chromosomal Karyotype of the Fetuses and Family Members

Amniotic fluid samples, umbilical cord blood samples, and peripheral blood samples of family members were subjected to conventional karyotyping because balanced rearrangements will escape SNP array detection (Levy and Wapner, 2018).

Pedigrees 1–6: The conventional G-banding analysis showed that the karyotypes of the fetuses and their parents were normal.

Pedigree 7: Although typical karyotypic analysis by G-banding might be able to delineate chromosomal aberrations greater than 5–10 Mb in size (Shaffer and Bejjani, 2004), the 8.7 Mb deletion of 21q21.1–21.2 was not identified in our study. Owing to the small size of chromosome 21, the deletion region could only be identified above 700-banded resolution, whereas the conventional amniotic karyotyping could only achieve 550-banded resolution at most. Therefore, the fetus (III:2, Figure 1), its elder brother (III:1, Figure 1), mother (II:2, Figure 1), and maternal grandmother (I:2, Figure 1) had normal karyotypes. Its father (II:1, Figure 1) and maternal grandfather (I:1, Figure 1) also had normal karyotypes.

**FIGURE 1:**
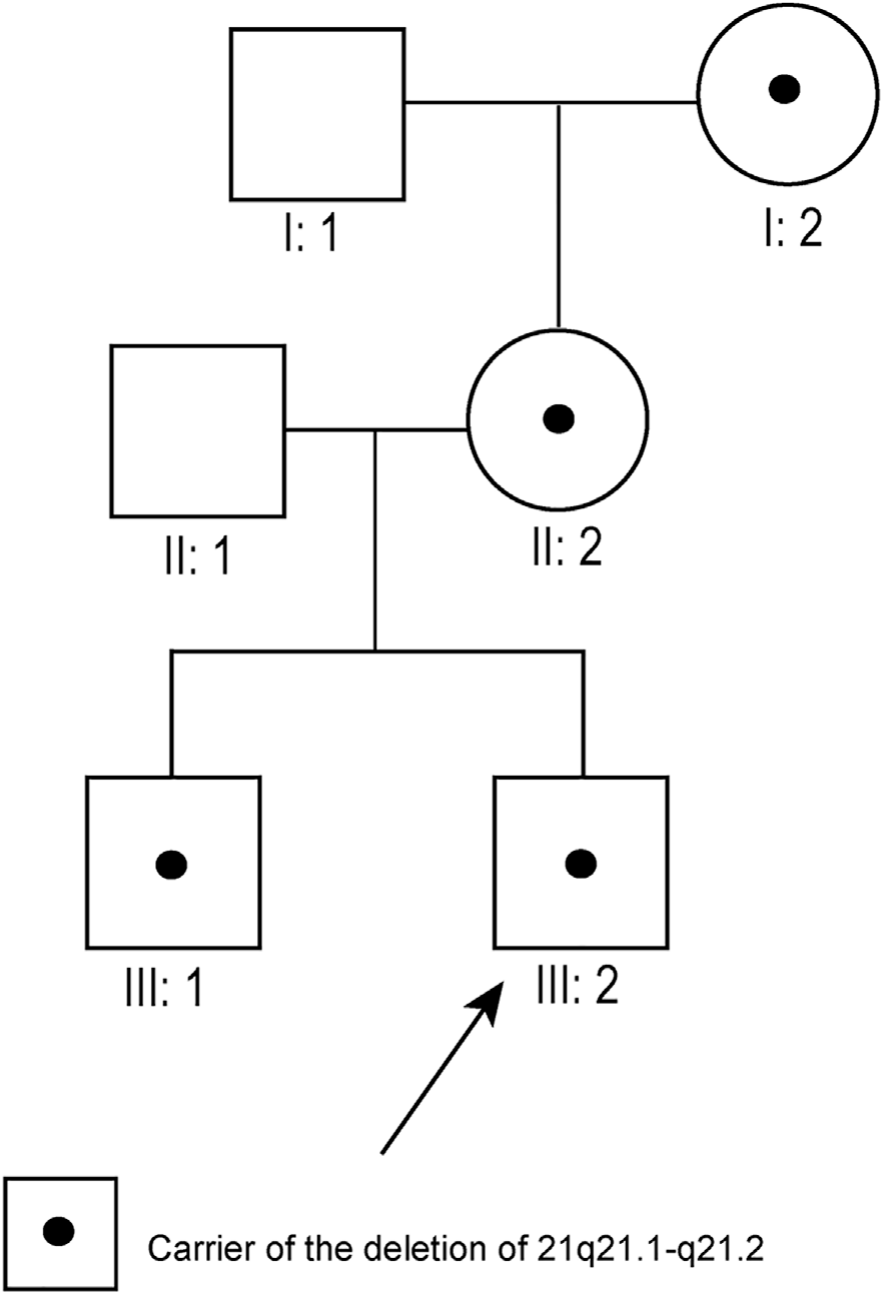
Pedigree diagram of pedigree 7 (arrow indicates the fetus). The fetus’s maternal grandmother, mother, and brother all carried the 21q21.1–21.2 deletion.

### Verification of SNP Array Results of the Fetuses and Family Members

**FIGURE 2:**
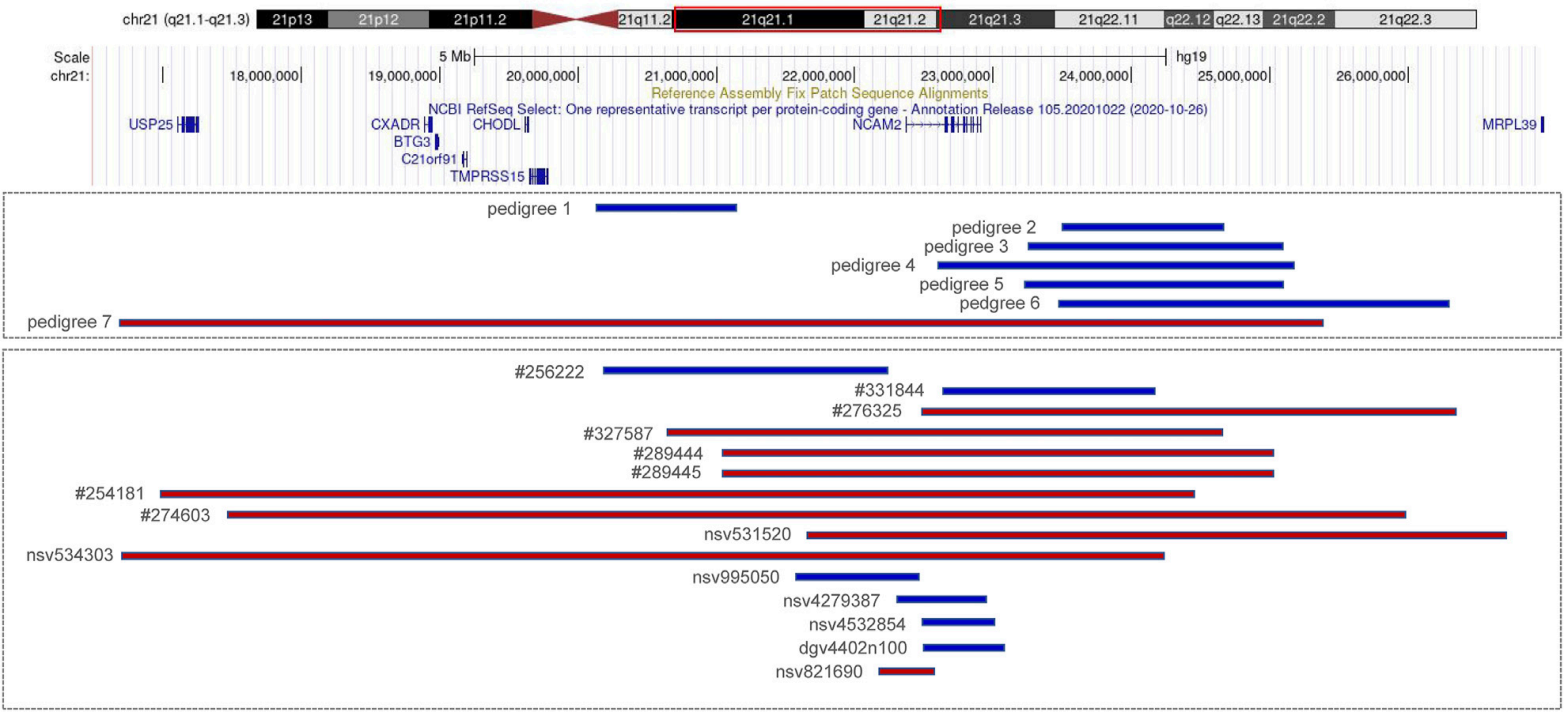
Copy number variations (CNVs) of 21q21.1–q21.2 (blue indicates duplication and red indicates deletion). Pedigrees 1–7 are from our cases; #256222, #331844, #276325, #327587, #289444, #289445, #254181, and #274603 are recorded in Decipher; nsv995050, nsv531520, and nsv534303 are recorded in dbVar; nsv4279387and nsv4532854 are recorded in gnomAD; dgv4402n100 and nsv821690 are recorded in DGV.

Pedigrees 1–6: The fetuses of six unrelated pedigrees carried the 21q21.1–q21.2 duplications, which were inherited from their mothers, and with the same coordinates and lengths as those of their mothers. The smallest duplication length was 1 Mb (chr21:20,195,657–21,199,532, hg19 build), and the largest was 2.7 Mb (chr21:23,573,580–26,310,725, hg19 build). The duplicated regions in pedigrees 1, 2, 3, 5, and 6 did not contain any protein-coding gene, and only the duplication in pedigree 4 contained the *NCAM2* gene (Table 1; Figure 2; Supplementary Figure S1). All fathers were also tested with SNP arrays, and the results were negative.

**TABLE 1:** Chromosomal aberrations of the fetuses in seven pedigrees.

Pedigree	Location (hg19)	Size (Mb)	Aberration type	Karyotype	Protein-coding gene content	Inheritance
Pedigree 1	chr21: 20,195,657–21,199,532	1	Duplication	46,XY	—	mat
Pedigree 2	chr21:23,573,580–24,697,989	1.1	Duplication	46,XX	—	mat
Pedigree 3	chr21: 23,288,789–25,106,099	1.8	Duplication	46,XY	—	mat
Pedigree 4	chr21: 22,734,409–25,148,429	2.4	Duplication	46,XX	NCAM2	mat
Pedigree 5	chr21:23,272,300–25,104,945	1.8	Duplication	46,XX	—	mat
Pedigree 6	chr21: 23,573,580–26,310,725	2.7	Duplication	46,XY	—	mat
Pedigree 7	chr21: 16,767,983–25,441,375	8.7	Deletion	46,XY	BTG3, C21orf91, CHODL, CXADR, NCAM2, TMPRSS15, USP25	mat

mat, Inherited from the mother.

Pedigree 7: SNP array results showed that the fetus (III:2, Figure 1) carried an 8.7 Mb deletion of chromosome 21q21.1–21.2 (chr21:16,767,983–25,441,375, hg19 build; Figure 2; Supplementary Figure S1), and the pedigree analysis found that the CNV was inherited from the mother with a normal phenotype (II:2, Figure 1). To obtain more genetic evidence, the elder brother and maternal grandparents of the fetus were also tested with SNP arrays. The extended analysis of the pedigree revealed that two other healthy members also carried the deletion, the elder brother, 3 years of age (III:1, Figure 1), and maternal grandmother, 41 years of age (I:2, Figure 1). In addition, the results of others (II:1 and I:1, Figure 1) in this pedigree were normal. Otherwise, the elder brother was found to carry another deletion, which was located on 5p15.33 (chr5:19:38,139–1,124,703, hg19 build) and was proven to be a *de novo* variation of uncertain significance (VUS) mutation. No other significant CNVs were found among the seven pedigrees.

### Prenatal and Postnatal Follow-Up Assessment

Pedigrees 1–6: No abnormalities were found during the second and third trimesters of pregnancy, except for the fetuses in pedigree 3 with pulmonary isolation. Three boys (fetuses of pedigrees 1, 3, and 6) and three girls (fetuses of pedigrees 2, 4, and 5) were born at full-term delivery. Now, the youngest individual is 2.5 years of age, the oldest is 3.5 years of age, and none of them show signs of developmental delay or intellectual disability based on child’s healthcare examination (Table 2).

**TABLE 2:** Clinical follow-up evaluation of 7 fetuses.

Fetus	Sex	At birth		Birth with other defects	Routine child healthcare (6 m, 12 m, 24 m)	Child healthcare by DDST		At study		
		Weight (kg) (%)	Length (cm) (%)			18 m	24 m	Age (m)	Weight (kg) (%)	Height (cm) (%)
1	Male	3.3 (46)	51 (72.2)	_	Pass	NA	NA	42	15 (42.5)	102 (70.6)
2	Female	3.3 (56)	50 (67.7)	_	Pass	Pass	NA	41	15.5 (63.2)	101 (73.4)
3	Male	3.05 (26.8)	50 (52.4)	Pulmonary sequestration	Pass	NA	NA	41	14.5 (33.7)	98 (34.3)
4	Female	3.3 (56)	50 (67.7)	_	Pass	NA	NA	37	14 (50.3)	95 (44.4)
5	Female	3.5 (71.6)	51 (83.9)	_	Pass	NA	NA	33	14 (67.3)	93 (53.5)
6	Male	3 (23.3)	48 (15.9)	_	Pass	NA	Pass	32	14 (60.0)	94 (60.3)
7	Male	2.35 (1.1)	48 (15.9)	_	Pass	NA	NA	8	8.5 (49.9)	70 (48.1)

m, months; NA, not available; percentile refers to WHO, Growth Charts.

Pedigree 7: Ultrasound and MRI examinations were performed at 32 weeks of gestation, and no further widening of the lateral ventricles was observed in both examinations (left: 14 mm, right: 14 mm, examined by ultrasound; left: 12.5 mm, right: 13.6 mm, examined by MRI). After genetic counseling, the pregnant woman and her husband chose to continue the pregnancy. A healthy boy was born by natural delivery at 39 gestational weeks, without any special facial features. The boy is 8 months old currently and does not have any abnormal phenotypes; moreover, the details of the child’s healthcare examination were normal (Table 2). His elder brother is 3 years of age and also does not show developmental delay or intellectual disability.

## Discussion

We reported seven fetuses carrying familial 21q21.1–21.2 aberrations. The fetuses of pedigrees 1, 2, 3, 4, 5, and 6 all carried a maternally inherited 21q21.1–q21.2 duplication ranging from 1 to 2.7 Mb (Table 1). There are few reports about whether the duplication of this region is benign or pathogenic. In public databases such as DGV, gnomAD, DECIPHER, dbVar, and ClinVar, several significant records of 21q21.1–q21.2 duplications were found, which partially overlapped with our cases, and they were analyzed (Figure 2; Table 3). Only three records were recorded in DGV (dgv4402n100) and the gnomAD database (nsv4279387, nsv4532854), but the frequency of copy number gains in the general population had not been described. In addition, a case with intellectual disability had been reported (DECIPHER, #256222), but there is no description about its inheritance and classification of pathogenicity. Another case (DECIPHER, #331844) was described as a likely benign variant with no abnormalities other than increased nuchal translucency. A VUS variant (nsv995050) was found in the dbVar and the CinVar database, and the major phenotype was developmental delay. Therefore, duplication of this region was considered a VUS in previous reports. The 21q21.1–q21.2 duplication in our study contained only one protein-coding gene, *NCAM2*, which is not predicted to be a triplosensitive gene. Jin et al. reported that a fetus and its mother both carried a 6.7 Mb duplication of 21q21.1–q21.2 including *NCAM2*, but the phenotype was normal. The region was significantly larger than that of our cases (ranging from position 18,981,715 to 25,707,009). This study also provided benign clinical evidence for partial duplication of 21q21.1–q21.2 in prenatal diagnosis (Jin et al., 2021). The rarity of gene content might be a major factor that makes these CNV gains, shown in this study, seem benign. In addition, position effects are one of the molecular mechanisms responsible for CNVs caused by genomic rearrangements resulting in phenotypes (Zhang et al., 2009). Whether the pathogenicity can change if the chromosomal duplication is not in its original position but translocates to another chromosome requires further study.

**TABLE 3:** Summary of patients harboring 21q21.1–q21.2 aberrations.

Patient database	Location (hg19)	Type	Size (Mb)	Protein-coding gene	Inheritance	Pathogenicity	Phenotypes
dbVar#nsv995050	chr21:21,601,231–22,573,421	Duplication	972 kb	NCAM2	Unknown	VUS	Developmental delay and/or other significant developmental or morphological phenotypes
Decipher#256222	chr21:20,063,479–22,274,948	Duplication	2.2 Mb	—	Inherited from a normal parent	/	Intellectual disability
Decipher#331844	chr21:22,782,651–24,339,651	Duplication	1.6 Mb	NCAM2	Inherited from the father	Likely benign	Increased nuchal translucency
Decipher#276325	chr21:22,434,634–26,315,434	Deletion	3.8 Mb	NCAM2	Inherited from the affected mother	/	Behavioral abnormality, delayed speech and language development
Decipher#327587	chr21:20,746,935–24,683,731	Deletion	3.9 Mb	NCAM2	Unknown	/	Overweight, recurrent otitis media, sandal gap, abnormal oral glucose tolerance, acanthosis nigricans, generalized non-motor (absence) seizure, generalized-onset seizure, simple febrile seizure, status epilepticus
Decipher#289444	chr21:21,044,211–25,051,262	Deletion	4.0 Mb	NCAM2	Unknown	VUS	Abnormal facial shape, short stature, intellectual disability
Decipher#289445	chr21:21,044,211–25,051,262	Deletion	4.0 Mb	NCAM2	Unknown	VUS	Intellectual disability
dbVar#nsv531520	chr21:21,699,837–26,771,050	Deletion	5.1 Mb	NCAM2	Inherited from the mother	Pathogenic	Abnormality of the skeletal system, cleft palate, global developmental delay
dbVar#nsv534303	chr21:16,714,035–24,198,636	Deletion	7.5 Mb	BTG3, C21orf91, CHODL, CXADR NCAM2, TMPRSS15, USP25	Unknown	Pathogenic	Oral cleft
Decipher#254181	chr21:16,992,255–24,898,237	Deletion	7.9 Mb	BTG3, C21orf91, CHODL, CXADR NCAM2, TMPRSS15, USP25	Inherited from the mildly affected father	/	Epicanthic folds, long and flat philtrum, high palate, low-set ears, global developmental delay, behavioral disorder
Decipher#274603	chr21:17,451,703–25,948,154	Deletion	8.5 Mb	BTG3, C21orf91, CHODL, CXADR NCAM2, TMPRSS15	Unknown	/	Almond-shaped eyes, hypotonia and joint laxity, global developmental delay, impaired social interactions

/, not provided by database.

The pathogenicity of the copy-number gain and copy-number loss might be quite different in the same region. A copy-number loss record involving *NCAM2* was found in the DGV database (nsv821690, Figure 2), but the frequency in the general population had not been described. Several cases of phenotypic abnormalities related to 21q21.1–21.2 deletions, and those highly overlapping with our case, were found in the public databases (Figure 2; Table 3). The sizes of these regions were approximately 4 Mb or greater. Three cases (Decipher#276325, Decipher#254181, and Decipher#274603), provided by Petit et al., their inheritance, and phenotypes had been described in detail (Petit et al., 2015). In five cases (Table 3), the deletion involved only *NCAM2*, and patients had abnormal phenotypes including those concerning intellectual disability, developmental delay, abnormal facial shape, and seizures. In two cases (nsv531520 and 534303), one was reported to have an abnormality of the skeletal system, cleft palate, and global developmental delay, and another had an oral cleft. They were all described as pathogenic, of which only one CNV (nsv531520) was inherited from the mother, but no information was provided about her phenotype. In summary, the 21q21.1–21.2 deletion was identified as likely pathogenic in previous reports. However, the 21q21.1–21.2 deletion in our study was not found to be associated with phenotypic consequences.

Previous and recent studies have revealed the important role of *NCAM2* in neurodevelopment (Sheng et al., 2019). In addition to *NCAM2*, there are other genes associated with clinical phenotypes in this region that deserve further analysis. This region contains seven protein-coding genes, namely, *BTG3*, *C21orf91*, *CHODL*, *CXADR*, *NCAM2*, *TMPRSS15*, and *USP25*. None of them are predicted to be haploinsufficient. Except for *C21orf91*, others are Online Mendelian Inheritance in Man (OMIM) genes. *BTG3* is a novel member of the PC3/BTG/TOB family of growth inhibitory genes (Yoshida et al., 1998) and is expressed in various human tissues. Further analysis in mice revealed that BTG3 is highly expressed in the ventricular zone of the developing central nervous system. *C21orf91* was described as having a role in defective DS neurogenesis (Li et al., 2016) and plays an important role in accurate oligodendroglial differentiation, affecting maturation capacity and axon myelination (Reiche et al., 2021). *CHODL* is a type-1A integral membrane protein and is preferentially expressed in the skeletal muscle, testis, brain, and lung (Weng et al., 2003). A recent study showed that the absence of *CHODL* leads to anatomical and functional defects of the neuromuscular synapse (Oprişoreanu et al., 2019). *CXADR* is expressed at increased levels during brain development and is considered a candidate gene in children with autism (Iourov et al., 2010). Patients carrying 21q21.1 microduplication (from 0.4 to 0.1 Mb) involving the *CXADR* gene have abnormal phenotypes such as developmental delay and intellectual disability (Li et al., 2018). *TMPRSS15* is a morbid gene, and loss-of-function variants are responsible for enterokinase deficiency (Wang et al., 2020). It is well known that *USP25* is widely expressed in the central nervous system and peripheral nervous system (Bosch-Comas et al., 2006). Recent studies have shown that USP25 plays a key role in microglial homeostasis reprogramming in Alzheimer’s disease and DS (Zheng et al., 2021). Therefore, *BTG3*, *C21orf91*, *CXADR*, *NCAM2*, and *USP25* might be involved in phenotypes based on their presumed or known biological functions.

The mechanism through which aberrations do not produce clinical phenotypes is unclear. Genetic counseling in this region has become challenging, owing to limited or conflicting associations with clinical phenotypes described in the published literature and public databases. Accordingly, our study provides benign evidence for accurate genetic counseling of 21q21.1–21.2 aberrations based on prenatal diagnosis.

## Data Availability

The datasets presented in this study can be found in online repositories. The names of the repository/repositories and accession number(s) can be found below: https://www.ebi.ac.uk/ena/browser/view/PRJEB47787.

## Supplementary Material

Supplementary Figure 1 Single-nucleotide polymorphism (SNP) array results for the fetuses. Pedigrees 1–6: 21q21.1–21.2 duplications, with fragment sizes in the following order: 1 Mb, 1.1 Mb, 1.8 Mb, 2.4 Mb, 1.8 Mb, and 2.7 Mb. Pedigree 7: 21q21.1–21.2 deletion; the fragment size was 8.7 Mb.
