# Supplementary figures and images for: Prenatal Diagnosis and Genetic Analysis of 21q21.1–q21.2 Aberrations in Seven Chinese Pedigrees
Source: Front Genet. 2021 Dec 21;12:731815. doi: 10.3389/fgene.2021.731815 (PMC8724545; doi:10.3389/fgene.2021.731815)

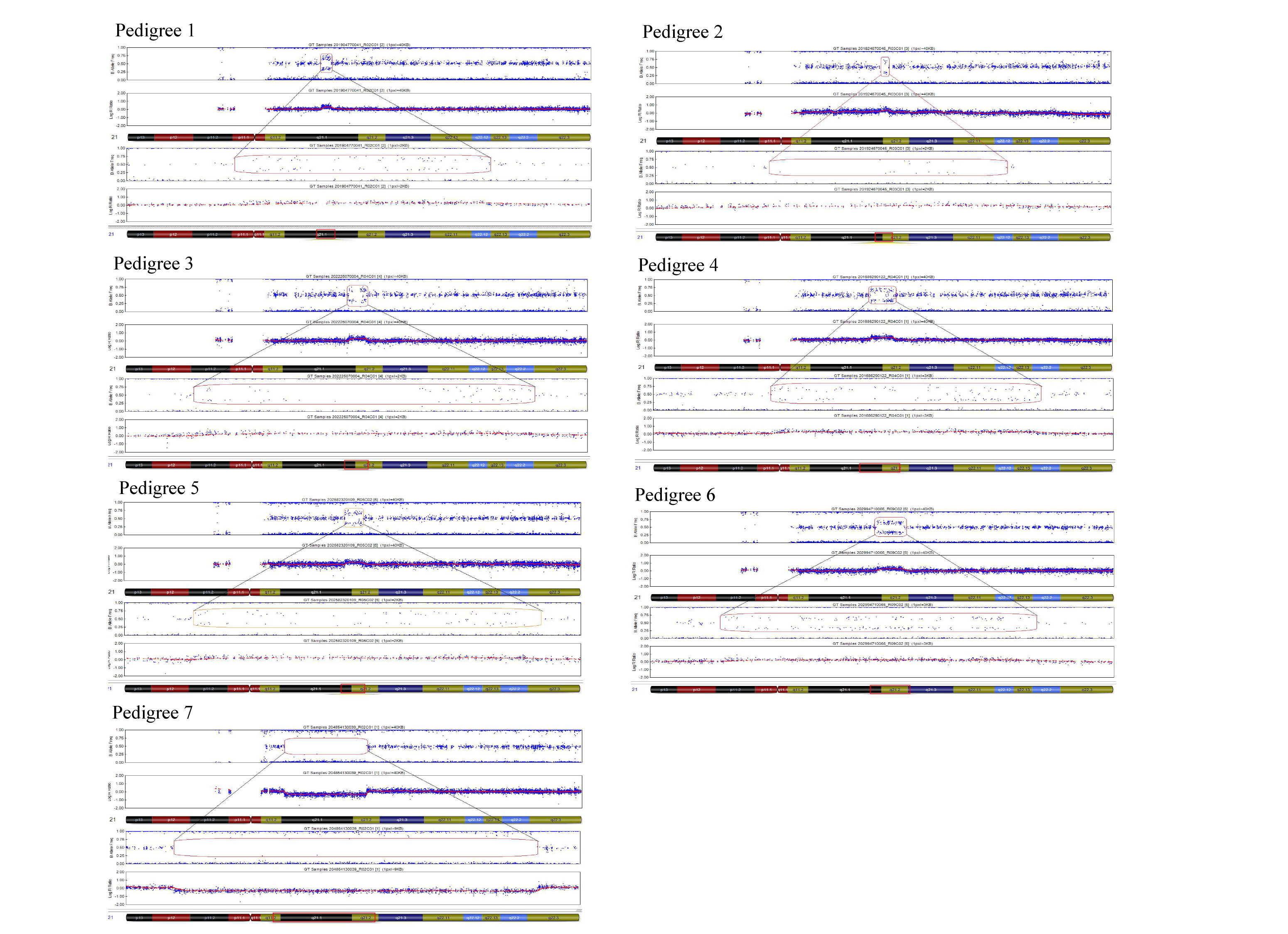

Supplement: Supplementary file 1 [file Figure3.TIF]
